# High Plasma Osteopontin Levels Are Associated with Serious Post-Acute-COVID-19-Related Dyspnea

**DOI:** 10.3390/jcm13020392

**Published:** 2024-01-10

**Authors:** Apostolos G. Pappas, Konstantinos Eleftheriou, Vassilios Vlahakos, Sophia F. Magkouta, Theofani Riba, Konstantina Dede, Rafaela Siampani, Steven Kompogiorgas, Eftychia Polydora, Athanasia Papalampidou, Natasa-Eleni Loutsidi, Nikolaos Mantas, Ekaterini Tavernaraki, Demetrios Exarchos, Ioannis Kalomenidis

**Affiliations:** 1 First Department of Critical Care and Pulmonary Medicine, “Evangelismos” General Hospital, School of Medicine, National and Kapodistrian University of Athens, 10676 Athens, Greecefanhriba@gmail.com (T.R.); konstadede@med.uoa.gr (K.D.); papalampidoua@hotmail.com (A.P.); ikalom@med.uoa.gr (I.K.); 2 “Marianthi Simou Laboratory”, First Department of Critical Care and Pulmonary Medicine, “Evangelismos” General Hospital, School of Medicine, National and Kapodistrian University of Athens, 10676 Athens, Greece; 3 Department of Pulmonary Medicine, “Evangelismos” General Hospital, 10676 Athens, Greece; 4 Hematology—Lymphomas Department and Bone Marrow Transplant Unit, “Evangelismos” General Hospital, 10676 Athens, Greece; natloutsidi@gmail.com; 5 Department of CT-MRI, “Evangelismos” General Hospital, 10676 Athens, Greecejimexarhos@yahoo.com (D.E.)

**Keywords:** osteopontin, long COVID, post-acute COVID-19, SARS-CoV-2, dyspnea, quality of life

## Abstract

COVID-19 survivors commonly report persistent symptoms. In this observational study, we investigated the link between osteopontin (OPN) and post-acute COVID-19 symptoms and lung functional/imaging abnormalities. We recorded symptoms and lung imaging/functional data from previously hospitalized COVID-19 patients, who were followed for 4–84 weeks (122 patients/181 visits) post-symptom onset at our outpatient clinic. Circulating OPN was determined using ELISA. Plasma OPN levels were higher in symptomatic patients (compared with the asymptomatic ones); those with dyspnea (compared with those without dyspnea);those with a combination of serious symptoms, i.e., the presence of at least one of the following: dyspnea, fatigue and muscular weakness (compared with those with none of these symptoms); and those with dyspnea and m-MRC > 1 (compared with those with m-MRC = 0–1). Plasma OPN levels were inversely correlated with EQ-VAS (visual analog scale of the EQ-5D-5L health-related quality-of-life questionnaire) values. High-resolution CT or diffusion lung capacity (DLCO) findings were not related to circulating OPN. In the multiple logistic regression, the presence of symptoms, dyspnea, or the combination of serious symptoms were linked to female gender, increased BMI and pre-existing dyspnea (before the acute disease), while increased plasma OPN levels, female gender and pre-existing dyspnea with m-MRC > 1 were independently associated with severe post-COVID-19 dyspnea (m-MRC > 1). Using a correlation matrix to investigate multiple correlations between EQ-VAS, OPN and epidemiological data, we observed an inverse correlation between the OPN and EQ-VAS values. Increased circulating OPN was linked to the persistence of severe exertional dyspnea and impaired quality of life in previously hospitalized COVID-19 patients.

## 1. Introduction

Early during the COVID-19 pandemic, it was noticed that patients recovering from acute disease may present with long-term symptoms and functional deficits. The term “long COVID syndrome” comprises abnormalities that persist for at least 3 months after the acute disease [1]. This heterogeneous condition impairs multiple organ systems [2], it may affect at least 10–30% of COVID-19 individuals [3,4] and it is characterized by the structural damage of vital organs [5]. Patients with severe insult from SARS-CoV-2 infection more commonly report persistent symptoms and may present with more severe forms of long COVID compared with those with mild initial infection [5,6,7]. Patients with long COVID often complain about fatigue, muscular weakness [8] and respiratory symptoms [9], and they exhibit impaired pulmonary function tests and abnormal chest CT imaging [10]. In a large cohort of hospitalized patients with COVID-19, it was demonstrated that up to 55% reported at least one symptom 2 years after the initial infection, with fatigue and psychological abnormalities being the most prevalent, while 14% of them exhibited impaired quality of life [11].

The underlying mechanisms of post-acuteCOVID-19 sequelae remain elusive [3,12]. Osteopontin (OPN) is a glycoprotein normally expressed in several tissues and is involved in a variety of human diseases [13], including severe influenza infection [14] and idiopathic pulmonary fibrosis [15,16]. In acute COVID-19, high circulating OPN levels have been associated with an increased risk of death or the need for mechanical ventilation [17] and increased levels of OPN at admission were shown to predict disease progression [18]. More specifically, it was recently described that high-OPN-expressing macrophages with pro-fibrotic properties were found in the bronchoalveolar lavage fluid and lung tissue obtained from patients with severe/critical COVID-19 [19].

The aim of this study was to investigate the potential link between OPN and post-acute COVID-19 symptoms and functional and imaging abnormalities. We hypothesized that increased circulating OPN levels are linked to the persistence of symptoms (including dyspnea), impaired quality of life, decreased oxygen diffusion lung capacity (DLCO) and extensive abnormal lesions in high-resolution chest computed tomography (HRCT).

## 2. Patients andMethods

This observational study was a sub-study of the Prospective Evangelismos Long COVID Study (PELCS), which enrolled 284 patients (previously hospitalized for acute COVID-19 at “Evangelismos” General Hospital, Athens, Greece, between 31 August 2020 and 17 September 2021). Patients were followed at the post-COVID-19 outpatient clinic of the “Evangelismos” General Hospital from 22 October 2020 to 28 September 2022. The inclusion criteria were the following: (a) age over 18 years and (b) hospitalization for SARS-CoV-2-related pneumonia with a positive PCR swab test at admission. The exclusion criteria were the following: (a) pregnancy, (b) transfer to another institution during acute COVID-19, (c) dementia and(d) inability to attain follow-up visits in the outpatient clinic. The study was conducted in line with the principles of the Declaration of Helsinki. All patients signed an informed consent form. Approval was granted by the Ethics Committee of the “Evangelismos” General Hospital (Athens, Greece), protocol number: 472/15-10-2020 (15 October 2020).

According to the PELCS protocol, a first follow-up visit was solicited at approximately 30 days after hospital discharge or earlier if clinically indicated. The initial evaluation included a medical history, physical examination, chest X-ray, and assessment of the quality of life using the modified medical research dyspnea scale (m-MRC) [7,11] and the European quality of life (EQ-5D-5L) self-assessed questionnaire [20]. For the purpose of the final analysis, the visual analog scale of the EQ-5D-5L score (EQ-VAS) was used [11] since it is characterized by a reliable discriminative capacity of patients with impaired quality of life due to long COVID syndrome [21]. A second visit was planned for approximately 60 days after the first one unless it was required earlier for clinical purposes. At that time, pulmonary function testing (PFT), including spirometry and measurement of lung volumes and DLCO, was performed. In addition, an HRCT was also ordered if the initial post-discharge chest X-ray and/or chest physical examination disclosed abnormal findings. Two senior chest radiologists graded HRCT images by consensus to determine the extent of lung abnormalities by calculating a “total lung severity score” (with values ranging from 0 to 20), as described elsewhere [22,23]. Further patient evaluations (with repetition of PFT and/or HRCT) were conducted for clinical purposesat the discretion of the supervising physician. Follow-up visits conducted in a period smaller than 90 days after the initial infection were considered “early visits” and those conducted in a period larger than 89 days post-symptom onset were considered “late visits”. Symptoms, m-MRC, EQ-5D-5L/EQ-VAS, PFT and imaging data were recorded.

In the context of PELCS, blood samples were obtained from the patients during visits that occurred between 23 April 2021 and 4 July 2022 at the outpatient clinic in order to investigate possible links between the levels of plasma soluble mediators and the post-discharge clinical/functional condition of the patients. Blood samples were centrifuged for 15 min at 1000 g/4 °C and plasma was obtained and stored at −80 °C. OPN levels were determined using a Human Osteopontin ELISA kit (catalog number DY 1433, R&D Systems, Minneapolis, MN, USA) [24].

### Statistical Analysis

Continuous variables are presented as mean ± standard error of the mean (SEM) if they were normally distributed and median (25–75 interquartile range (IQR)) or median (5–95 whisker plots) if they were not normally distributed. Categorical variables are presented as frequencies and percentages. Comparisons between the OPN levels of different groups were made using the Mann-Whitney Utest. Comparisons between categorical variables were conducted using Fisher’s exact test. In order to identify independent predictors for the development of persistent post-acute COVID-19 symptomatology, two multiple logistic regression models were developed; the plasma OPN level was considered as the independent variable and any symptom and dyspnea were considered as the dependent variables. Age, gender, body mass index (BMI), Charlson co-morbidity index (CCI), non-severe acute COVID-19 according to WHO (i.e., patients with no need for supplemental oxygen during hospitalization) and pre-existing dyspnea were treated as covariates. In order to identify independent predictors for the development of serious post-acute COVID-19 sequelae, two multiple logistic regression models were developed; the plasma OPN level was considered as the independent variable and a serious symptom combination (presence of at least one of the following: dyspnea, fatigue and muscular weakness) or dyspnea with m-MRC > 1 were considered as the dependent variables. Age, gender, BMI, CCI, non-severe acute COVID-19 according to WHO and pre-existing exertional dyspnea (m-MRC > 1) were treated as covariates. For each regression model, the area under the receiver operating characteristic (AU-ROC) curve was calculated. Multiple correlations were investigated using a correlation matrix and Spearman’s coefficient was calculated. All tests were two-tailed and *p*-values <0.05 were considered significant. Statistical analysis was performed using Graphpad Prism Software (Version 9, San Diego, CA, USA).

## 3. Results

### 3.1. PatientSamples

We analyzed data from 122 patients, who were followed at the post-acuteCOVID-19 outpatient clinic 4–84 weeks after symptom onset (total 181 visits) and from whom blood samples were obtained. During the study period, 74 patients were evaluated once, 38 patients were evaluated twice, 9 patients were evaluated three times and 1 patient was evaluated four times. A plasma sample was obtained during every one of the above visits. The mean ± SEM age of the patients was 59.5 ± 1.1 years, their median (IQR) BMI was 28 (25.9–32) and their median (IQR) CCI was 2 (1–3). A total of 84 (69%) were male and 67 (55%) were smokers. During the acute disease, 98 (80.3%) required supplemental oxygen and 5 (4%) were intubated.

### 3.2. Clinical, Functional and HRCT Post-Acute COVID-19 Abnormalities

The patients’ symptoms at early post-discharge (within 89 days after the onset of the acute disease) or late post-discharge (90 days or more after the acute disease onset) visits are shown in Table 1. The median (IQR) EQ-VAS scores were 80 (70–95) and 80 (70–90) when early visits and late visits were considered, respectively. PFTs from 95 patients (a total of 120 tests, with 25 patients having two tests) were conducted at a median (IQR) of 185 (124–227) days after symptom onset. Only one patient underwent PFTs before 90 days post-symptom onset. The mean ± SEM of the total lung capacity (TLC) was 95 ± 2.3% of the predicted value. The median (IQR) forced expiratory volume for the first second (FEV1) was 105 (94–113)% and the median (IQR) forced vital capacity (FVC) was 103 (94–113)% of the predicted value. The median (IQR) FEV1/FVC ratio was 81 (79–84) and the median (IQR) DLCO was 78 (68–84)% of the predicted value. The TLC, FVC, FEV1 and DLCO values <80% of the predicted value were observed in 9.5%, 4.5%, 2.8% and 54% of patients, respectively. A small minority of patients (2.75%) presented FEV1/FVC values < 0.7. A total of 102 HRCT scans from 88 patients (12 patients had two scans and one patient had three scans) were conducted. Seven patients were scanned in a period shorter than 90 days post-symptom onset; the median (IQR) total lung severity score (HRCT score) was 2.5 (0.5–4.75) and all seven patients exhibited residual abnormalities on the HRCT images. The rest of the 81 patients were evaluated later than 89 days after symptom onset. Their median (IQR) HRCT score was 0.75 (0–2.5), with 73% of them having residual HRCT abnormalities.

### 3.3. Link between Clinical, Functional and HRCT Post-Acute COVID-19 Abnormalities and Plasma OPNLevels

Analyses were performed either using OPN levels from samples obtained during all visits (181 samples from 122 patients) or those from samples obtained during late visits, i.e., those that occurred beyond 89 days from the initial presentation of the acute disease (155 samples from 96 patients), which was the period used for the definition of “Long COVID” [1].

**Figure 1:**
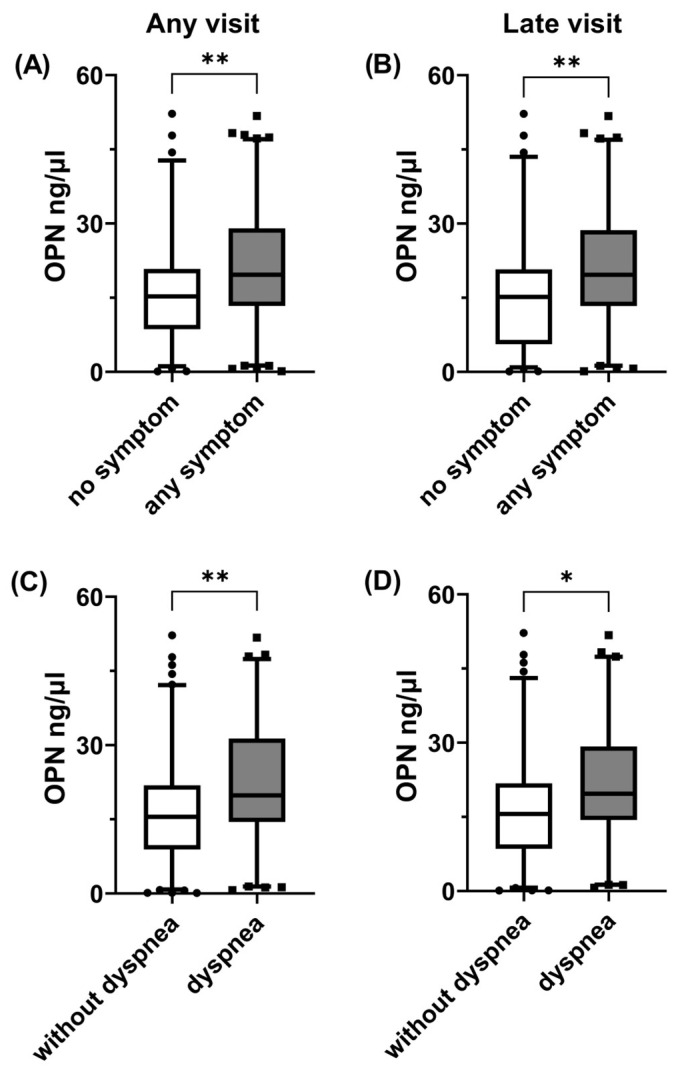
Increased plasma OPN levels were associated with long COVID symptoms in patients previously hospitalized for COVID-19. OPN levels from 181 plasma samples obtained during follow-up visits from 122 previously hospitalized COVID-19 patients were compared in the absence and presence of any symptom at any visit, n = 74 vs. n = 107 (**A**); any symptom at visits occurred 90 days or more (late visits) post-symptom onset, n = 67 vs. n = 87 (**B**); dyspnea at any visit, n = 102 vs.n = 79, (**C**); and dyspnea at late visits, n = 91 vs. n = 63 (**D**). Data are presented as 5–95 whisker plots with median; * *p* < 0.05, ** *p* < 0.01. OPN: osteopontin.

**Figure 2:**
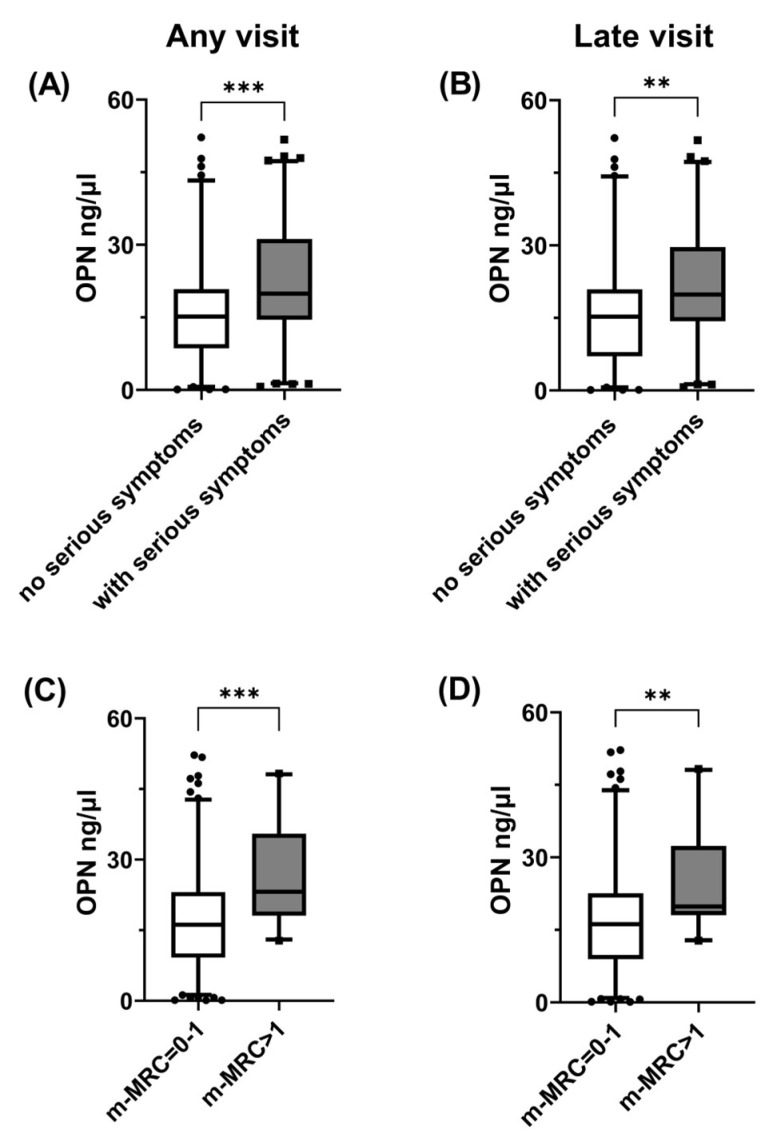
Increased plasma OPN levels were associated with severe post-acute COVID-19 sequelae.OPN levels from 181 plasma samples obtained during follow-up visits from 122 previously hospitalized COVID-19 patients were compared in the absence and presence of a serious symptom combination (defined as at least one of the following: dyspnea, fatigue and muscular weakness) at any visit, n = 89 vs. n = 92 (**A**), or at visits occurred 90 days or more (late visits) post-symptom onset, n = 80 vs. n = 74 (**B**); dyspnea where m-MRC > 1 at any visit, n = 146 vs. n = 29 (**C**); and dyspnea where m-MRC > 1 at late visits, n = 126 vs. n = 23 (**D**). Data are presented as 5–95 whisker plots with median, ** *p* < 0.01, *** *p* < 0.001. OPN: osteopontin, m-MRC: modified Medical Research Council dyspnea scale.

Plasma OPN levels were significantly higher in patients who reported at least one post-COVID-19 symptom compared with asymptomatic ones, either when the entire study population was considered (Figure 1A) or when only samples obtained during late visits were analyzed (Figure 1B). Similarly, patients who reported dyspnea had increased plasma OPN levels compared with patients without dyspnea, either when samples were obtained during all follow-up visits (Figure 1C) or only samples from late visits (Figure 1D) were analyzed. We then created a serious symptom combination, which included at least one of the following: dyspnea, fatigue and muscular weakness. The samples from patients with this symptom combination were characterized by higher plasma OPN levels compared with those obtained from patients with none of these symptoms, either when the entire population or the subset evaluated at late visits were considered (Figure 2A,B). In contrast, OPN levels did not differ among samples obtained from patients with or without fatigue or muscular weakness when any of these symptoms were evaluated separately When looking for a possible link between OPN and the severity of dyspnea, we observed that plasma OPN levels were higher in samples obtained from patients with m-MRC > 1 compared with those with m-MRC = 0–1 when the entire population was considered (Figure 2C). When the analysis was restricted only to late visits (Figure 2D), the samples from patients with m-MRC > 1 had higher OPN levels compared with those from patients with m-MRC = 0–1.

**Figure 3:**
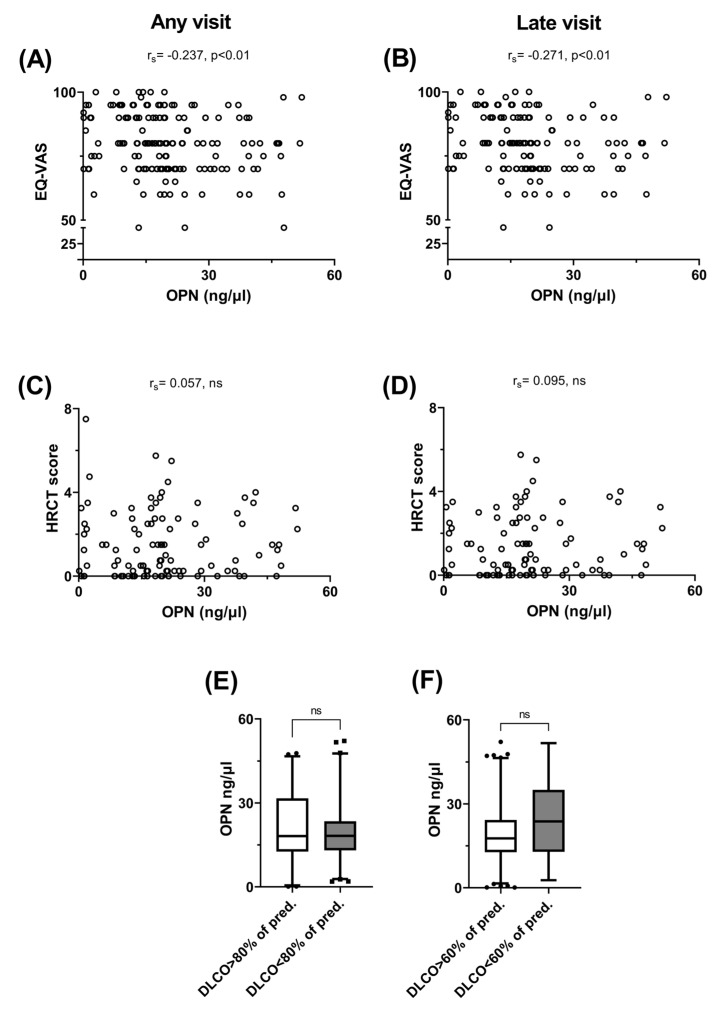
Increased plasma OPN levels were associated with impaired quality of life, but not with the extent of HRCT abnormalities and decreased DLCO in patients previously hospitalized for COVID-19. The correlation between plasma OPN levels from 122 patients and EQ-VAS score or HRCT findings was investigated. Scattergram and correlation between OPN levels and EQ-VAS score at any follow-up visit (pairs = 157) (**A**). Scattergram and correlation between OPN levels and EQ-VAS score at visits occurred 90 days or more (late visits) post-symptom onset (pairs = 133) (**B**). Scattergram and correlation between OPN levels and HRCT score at any visit (pairs = 102) (**C**) and late follow-up visits (pairs = 95) (**D**). Plasma OPN levels from patients with DLCO ≥ 80% or <80% (n = 55 vs. n = 65) of the predicted value (**E**). Plasma OPN levels from patients with DLCO ≥ 60% or <60% (n = 107 vs.n = 13) of the predicted value (**F**). Data are presented as 5–95 whisker plots with median. EQ-VAS: visual analog scale score of EQ-5D-5L, EQ-5D-5L: European quality of life self-assessed questionnaire, HRCT: high-resolution chest computed tomography, DLCO: diffusing lung capacity for carbon monoxide, rs: Spearman’s coefficient, ns: non-significant.

Having demonstrated the association between higher OPN levels and persistent symptoms, we aimed to investigate any possible link between circulating OPN and the overall quality of life (Figure 3A,B). Indeed, we observed an inverse correlation between the EQ-VAS score and plasma OPN levels, both when the entire study population was considered (Figure 3A) and when only late visits were analyzed (Figure 3B). Next, we looked for a possible link between the HRCT severity lung score and plasma OPN. It was observed that there was no correlation between the HRCT score and circulating OPN, both when considering all scans (Figure 3C) and when evaluating scans obtained beyond 89 days post-symptom onset (Figure 3D). Since a link with severe dyspnea was already established, we sought to determine whether decreased DLCO and OPN were related. Although OPN did not differ in samples obtained from patients with DLCO > 80% of the predicted value and those with DLCO < 80% (Figure 3E), we observed that patients with severely impaired oxygen diffusion capacity (DLCO < 60% of predicted value) had increased plasma OPN levels compared with patients with DLCO > 60% of the predicted value (Figure 3F). However, this difference was not statistically significant. Furthermore, we did not identify any link between the OPN levels and the TLC, FVC and FEV1 values.

### 3.4. Multiple Analyses

**Figure 4:**
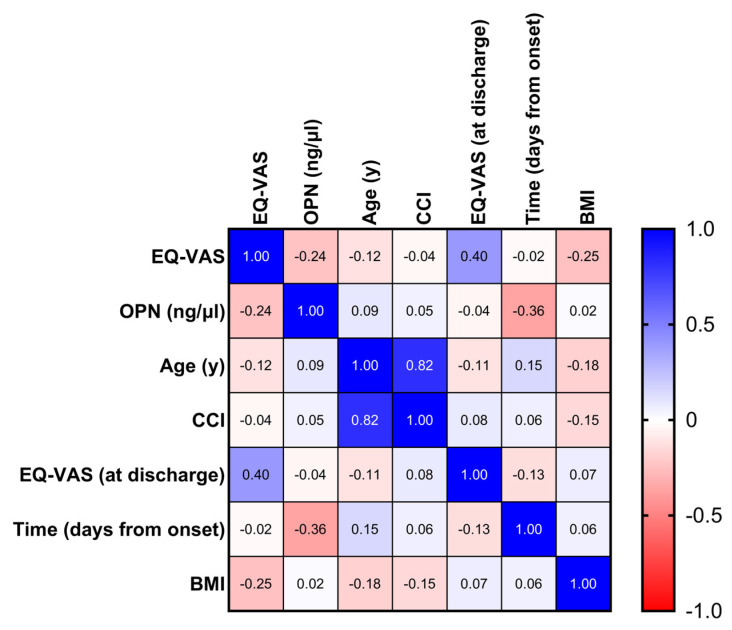
Increased plasma OPN levels, higher BMI and decreased EQ-VAS score at hospitaldischarge were associated with impaired quality of life. Multiple correlations were calculated (correlation matrixheat map) between patients’ clinical characteristics, EQ-VAS score at any visit and plasma OPN levels obtained from 122 patients during follow-up visits to identify various parameters linked to impaired quality of life after hospitalization for acute COVID-19. Significant correlations were the following: (a) EQ-VAS: OPN (*p* < 0.01), (b) EQ-VAS: EQ-VAS at discharge (*p* < 0.001), (c) EQ-VAS: BMI (*p* < 0.01), (d) OPN: days from symptom onset (*p* < 0.001), (e) age: CCI (*p* < 0.001), (f) age: BMI (*p* < 0.05) and(g) CCI: BMI (*p* < 0.05). For all correlations, Spearman’s coefficient was calculated. EQ-VAS: visual analog scale score of EQ-5D-5L, EQ-5D-5L: European quality of life self-assessed questionnaire, OPN: osteopontin, CCI: Charlson comorbidity index, BMI: body mass index.

We developed multiple logistic regression models to investigate whether plasma OPN levels were independently associated with the presence of any symptom, the presence of dyspnea, the presence of the combination of serious symptoms or the presence of dyspnea with m-MRC > 1 in patients previously hospitalized for COVID-19 (Table 2 and Table 3). We observed that female gender and pre-existing dyspnea (dyspnea that was reported before the onset of acute COVID-19) were linked to the presence of any symptom or dyspnea (Table 2). The presence of any dyspnea, fatigue or muscular weakness was linked to female gender and BMI > 30, while severe post-COVID-19 exertional dyspnea with m-MRC > 1 was associated with high plasma OPN, female gender and the presence of severe exertional dyspnea (m-MRC > 1) before the onset of the acute disease (Table 3). We then created a correlation matrix in order to investigate multiple correlations between circulating OPN, patients’ characteristics and impaired quality of life. Although the plasma OPN levels decreased significantly over time, we observed an inverse correlation between the OPN and EQ-VAS values (Figure 4). Other parameters significantly associated with a decreased EQ-VAS score were the lower EQ-VAS score recorded at hospital discharge and higher BMI. Conversely, age, CCI and days from symptom onset were not correlated with EQ-VAS score.

## 4. Discussion

We investigated the association of circulating OPN with persistent symptomatology and functional/imaging abnormalities in 122 previously hospitalized COVID-19 patients (>80% of whom required supplemental oxygen during hospitalization) who were followed up at an outpatient clinic for 4–84 weeks after the onset of symptoms. The main findings of our study were the following: (a) The increased plasma OPN levels were higher in samples obtained from symptomatic patients (compared with asymptomatic ones); from those with dyspnea (compared with those without dyspnea); from patients with a combination of serious symptoms, including dyspnea and/or fatigue and/or muscular weakness (compared with those without any of these symptoms); and from those with dyspnea with m-MRC > 1 (compared with those with m-MRC = 0–1). These differences were significant whether all visits or those that occurred beyond 89 days post-symptom onset were considered. (b) Plasma OPN levels were inversely correlated with EQ-VAS (visual analog scale of EQ-5D-5L) values when all or late visits were considered. (c) We did not identify any significant link between the circulating OPN and imaging or lung functional abnormalities. (d) In the multiple logistic regression analyses, the presence of symptoms, dyspnea or the combination of serious symptoms were linked to previously established risk factors, including female gender, increased BMI and pre-existing dyspnea (before the acute disease), while increased plasma OPN levels, female gender and pre-existing dyspnea with m-MRC > 1 were independently associated with severe post-COVID-19 dyspnea (m-MRC > 1). (e) Using a correlation matrix to investigate multiple correlations between circulating OPN, patients’ characteristics and impaired quality of life, circulating OPN was inversely correlated with impaired quality of life (decreased EQ-VAS score) during follow-up visits, despite the fact that the OPN levels were decreasing significantly over time. To our knowledge, this is the first study that demonstrated a link between circulating OPN and post-acute COVID-19 sequelae. We observed an association between plasma OPN levels and persistent symptomatology, as well as impaired quality of life. This link was also evident when observations made beyond 3 months after the onset of the acute illness were analyzed. OPN levels were independently associated with severe post-COVID-19-related dyspnea (m-MRC > 1), even when they were adjusted against previously recognized risk factors for long COVID (age, female gender, co-morbidities, pre-existing severe dyspnea, severity of the acute disease and obesity). These findings further expand previous reports implicating OPN with the development of pulmonary fibrotic lesions [19] and an increased risk for dismal outcomes [17,18] in patients with severe acute COVID-19. These observations come alongside accumulating evidence of pro-inflammatory cytokine over-expression (IL-1β, IL-6, TNF-a, interferons) in patients with long COVID [6,25,26].

The observation that dyspnea, fatigue and psychological symptoms were the most common patient complaints is in line with larger previously published studies [11,25]. Similar to previous reports [27], we observed that more than half of our patients had abnormal DLCO values and imaging findings beyond 3 months after the initial disease. However, neither DLCO values nor lung imaging abnormalities were related to the plasma OPN levels. Although these observations could be partly attributed to the small sample size, they are in line with previous reports [28] stating that new-onset dyspnea is rarely associated with fibrotic lesions on HRCT and impaired diffusion capacity. Furthermore, our findings do not support the possibility that the link between OPN and severe dyspnea is mediated by the pro-fibrotic effect of OPN [19] and further imply that OPN may lead to the development of extra-pulmonary pathology (e.g., muscle wasting) contributing to dyspnea [6]. Interestingly, OPN was shown to play a central role in experimental models of muscle injury/regeneration [29]. However, this is merely speculative and requires further research. Long-COVID-related symptoms were also found to be associated with established risk factors, including female gender, increased BMI and poorly controlled pre-existing disease (in our study this was demonstrated by the presence of pre-existing dyspnea) [25,30,31]. On the other hand, in contrast to previous investigations, older age and multiple co-morbidities (as expressed using the Charlson comorbidity index) were not independently related to persistent symptomatology, which might havepossibly beenrelated to either the small cohort size or the specific characteristics of the patients included in our study.

The limitations of the present study were the following: (a) This was a single-center study that did not include non-hospitalized patients with COVID-19. (b) Certain patients (especially those who improved over time) were lost from follow-up and this might have led to an over-estimation of the prevalence of severe symptoms. (c) Specific treatments for acute COVID-19 that provoke muscle wasting (e.g., corticosteroids [11]) were not evaluated in the multiple analyses. It should, however, be noted that in our practice, low-dose and low-duration steroid therapy was given only to patients with respiratory failure during the course of the acute disease [32]. (d) The fact that each follow-up visit and the corresponding blood sample were analyzed as separate observations might have led to the over-representation of the patients who had multiple follow-up visits. On the other hand, the inclusion of multiple follow-up visits permitted us to extend our observations to a period of up to 84 weeks and to identify patients who developed post-acute COVID-19 sequelae beyond 3 months after the initial illness. Our observations were also powered by the use of multiple regression and correlation models in order to identify circulating OPN as a predictor of severe dyspnea and impaired quality of life.

## 5. Conclusions

In conclusion, we demonstrated that increased circulating OPN is associated with persistent post-acute COVID-19 symptoms (mainly severe dyspnea) and impaired quality of life in patients previously hospitalized for acute COVID-19. Therefore, OPN might further be investigated as a potential biomarker of post-acute COVID-19 sequelae. In addition, our findings pave the way for further research in order to identify possible mechanisms that implicate OPN in post-acuteCOVID-19 dyspnea.

## Figures and Tables

**Table 1:** Post-acute COVID-19 symptoms of previously hospitalized patients at early and late time points after the initial illness.

	Early Visits	Late Visits	*p*-Value
Asymptomatic	25.93%	43.51%	0.1
Dyspnea	59.26%	40.91%	0.09
Dyspnea with m-MRC > 1	25%	15.97%	0.4
Cough	11.54%	8.44%	0.7
Fatigue	42.31%	23.87%	0.06
Muscular weakness	26.92%	13.55%	0.1
Myalgia/arthralgia	11.54%	5.81%	0.4
Weight loss > 4 kg	15.38%	9.68%	0.5
Dizziness/vertigo	7.69%	6.45%	0.7
Tremor	3.85%	1.95%	0.6
Numbness	0%	1.3%	0.9
Headache	7.69%	3.87%	0.3
Fever	0%	0.65%	0.9
Sweating	7.69%	5.84%	0.7
Gastrointestinal symptoms	0%	2.58%	0.9
Hairloss	11.54%	10.32%	0.7
Rash	0%	2.58%	0.9
Anosmia/ageusia	7.69%	1.3%	0.1
Palpitation	7.69%	5.16%	0.6
Psychological symptoms	23.08%	19.48%	0.4
Other	0%	4.52%	0.6

Symptoms of 122 patients during 4–84 weeks after the onset of acute disease (total 181 visits). Early follow-up visits are considered those that occurred within 89 days after acute COVID-19 onset (27 visits/27 patients) and late visits are considered those that occurred 90 days or more after the initial illness (154 visits/95 patients). Percentages correspond to symptoms reported by a patient during a visit. m-MRC: modified Medical Research Council dyspnea scale, EQ-VAS: visual analog scale of EQ-5D-5Lscore,EQ-5D-5L: European quality of life self-assessed questionnaire.

**Table 2:** Female gender and pre-existing dyspnea were associated with persistent symptomatology in patients previously hospitalized for acute COVID-19.

		Dependent Variable: Any Symptom			Dependent Variable: Dyspnea		
Parameter Estimates	Variable	Adjusted OR	95% CI	*p*- Value	Adjusted OR	95% CI	*p*- Value
**β0**	Intercept	7.56	0.65–97.36	0.11	2.32	0.15–35.5	0.5
**β1**	OPN (ng/μl)	1.02	0.99–1.05	0.13	1.02	0.99–1.06	0.1
**β2**	Age(y)	0.97	0.93–1.006	0.1	0.97	0.92–1.01	0.1
**β3**	Male gender	0.31	0.12–0.77	**0.01**	0.39	0.16–0.94	**0.03**
**β4**	BMI ≥ 30 (kg/m^2^)	1.76	0.84–3.77	0.14	1.43	0.63–3.28	0.4
**β5**	CCI > 2	0.97	0.36–2.65	0.95	1.27	0.41–4.12	0.7
**β6**	Non-severe acute illness	0.87	0.35–2.14	0.76	0.6	0.21–1.65	0.3
**β7**	Pre-existing dyspnea	10.26	4.07–30.07	**<0.001**	17.64	7.47–46.07	**<0.001**

Two multiple logistic regression models were developed using patient characteristics and measurements obtained during follow-up visits. Plasma OPN levels were considered as the independent variable and any symptom or dyspnea were considered as the dependent variables. Age, gender, BMI, CCI, COVID-19 severity (according to WHO) during hospitalization and the presence of dyspnea before hospitalization for COVID-19 were treated as covariates. AU-ROC curve for the first model was 0.81 (95% CI = 0.74–0.87, *p* < 0.001) and for the second one was 0.85 (95% CI = 0.76–0.91, *p* < 0.001). Estimates are presented as adjusted odds ratio (OR) and *p*-values <0.05 were considered significant. OPN: osteopontin, BMI: body mass index, CCI: Charlson comorbidity index, non-severe acute illness (WHO): no need for supplemental oxygen during hospitalization, WHO: World Health Organization, 95% CI: 95% confidence interval, AU-ROC curve: area under the receiver-operating characteristic curve.

**Table 3:** Higher circulating OPN levels, female gender, higher BMI and pre-existing exertional dyspnea (m-MRC > 1) are associated with serious post-acute COVID-19 sequelae during follow-up visits after hospitalization for acute disease.

		*Dependent Variable: Serious Symptom Combination*			*Dependent Variable: Dyspnea with m-MRC > 1*		
Parameter Estimates	Variable	Adjusted OR	95% CI	*p*-Value	Adjusted OR	95% CI	*p*-Value
**β0**	Intercept	4.08	0.41–43.45	0.23	0.04	0.001–1.17	0.08
**β1**	OPN (ng/μl)	1.02	0.99–1.05	0.22	1.05	1.004–1.09	**0.03**
**β2**	Age(y)	0.97	0.93–1.01	0.11	1.01	0.95–1.08	0.8
**β3**	Male gender	0.2	0.09–0.41	**<0.001**	0.3	0.098–0.86	**0.03**
**β4**	BMI ≥ 30 (kg/m^2^)	2.21	1.09–4.6	**0.03**	1.69	0.57–5.19	0.3
**β5**	CCI > 2	2.19	0.83–6.01	0.12	1.28	0.29–5.91	0.7
**β6**	Non-severe acute illness	0.79	0.33–1.83	0.59	1.53	0.43–4.97	0.5
**β7**	Pre-existing m-MRC > 1	2.84	0.67–15.08	0.18	71.22	10.5–1517	**<0.001**

Two multiple logistic regression models were developed using patient characteristics and measurements obtained during follow-up visits. Plasma OPN levels were considered as the independent variable and serious symptom combination or m-MRC > 1 were considered as the dependent variables. Age, gender, BMI, CCI, COVID-19 severity (according to WHO) during hospitalization and pre-hospitalization dyspnea with m-MRC > 1 were treated as covariates. AU-ROC curve for the first model was 0.76 (95% CI = 0.68–0.84, *p* < 0.001) and for the second one was 0.85 (95% CI = 0.75–0.94, *p* < 0.001). Estimates are presented as adjusted odds ratio (OR) and *p*-values <0.05 were considered significant. Serious symptom combination is defined as the presence of at least one of the following: dyspnea, fatigue and muscular weakness. OPN: osteopontin, m-MRC: modified Medical Research Council dyspnea scale, BMI: body mass index, CCI: Charlson comorbidity index, non-severe acute illness (WHO): no need for supplemental oxygen during hospitalization, WHO: World Health Organization, 95% CI: 95% confidence interval, AU-ROC curve: area under the receiver-operating characteristic curve.

## Data Availability

Data are available upon contact with the corresponding author.

## Abbreviations

OPN: osteopontin, COVID-19: coronavirus disease 2019, SARS-CoV-2: severe acute respiratory syndrome coronavirus 2, m-MRC: modified Medical Research Center dyspnea scale, EQ-5D-5L: European quality of life self-assessed questionnaire, EQ-VAS: visual analog scale of EQ-5D-5L. HRCT: high-resolution computed tomography, BMI: body mass index, CCI: Charlson comorbidity index, ELISA: enzyme-linked immunosorbent assay.
